# Quantitative EEG Markers in Mild Cognitive Impairment: Degenerative versus Vascular Brain Impairment

**DOI:** 10.1155/2012/917537

**Published:** 2012-07-26

**Authors:** D. V. Moretti, O. Zanetti, G. Binetti, G. B. Frisoni

**Affiliations:** Centro San Giovanni di Dio Fatebenefratelli, TRccs, 25125 Brescia, Italy

## Abstract

We evaluated the relationship between brain rhythmicity and both the cerebrovascular damage (CVD) and amygdalohippocampal complex (AHC) atrophy, as revealed by scalp electroencephalography (EEG) in a cohort of subjects with mild cognitive impairment (MCI). All MCI subjects underwent EEG recording and magnetic resonance imaging. EEGs were recorded at rest. Relative power was separately computed for delta, theta, alpha1, alpha2, and alpha3 frequency bands. In the spectral band power the severity of CVD was associated with increased delta power and decreased alpha2 power. No association of vascular damage was observed with alpha3 power. Moreover, the theta/alpha1 ratio could be a reliable index for the estimation of the individual extent of CV damage. On the other side, the group with moderate hippocampal atrophy showed the highest increase of alpha2 and alpha3 power. Moreover, when the amygdalar and hippocampal volumes are separately considered, within amygdalohippocampal complex (AHC), the increase of theta/gamma ratio is best associated with amygdalar atrophy whereas alpha3/alpha2 ratio is best associated with hippocampal atrophy. CVD and AHC damages are associated with specific EEG markers. So far, these EEG markers could have a prospective value in differential diagnosis between vascular and degenerative MCI.

## 1. Introduction

Mild cognitive impairment (MCI) is a clinical state intermediate between elderly normal cognition and dementia that affects a significant amount of the elderly population, featuring memory complaints and cognitive impairment on neuropsychological testing, but no dementia [1–3].

The hippocampus is one of the first and most affected brain regions impacted by both Alzheimer's disease and mild cognitive impairment (MCI; [4–9]). In mild-to-moderate Alzheimer's disease patients, it has been shown that hippocampal volumes are 27% smaller than in normal elderly controls [10, 11], whereas patients with MCI show a volume reduction of 11% [11]. So far, from a neuropathological point of view, the progression of disease from MCI state to later stages seems to follow a linear course. Nevertheless, there is some evidence from functional [12–14] and biochemical studies [15] that the process of conversion from nondemented to clinically evident demented state is not so linear. Recent fMRI studies have suggested increased medial temporal lobe (MTL) activations in MCI subjects versus controls, during the performance of memory tasks [16, 17]. Nonetheless, fMRI findings in MCI are discrepant, as MTL hypoactivation similar to that seen in AD patients [18] has also been reported [19]. Recent postmortem data from subjects—who had been prospectively followed and clinically characterized up to immediately before their death—indicate that hippocampal choline acetyltransferase levels are reduced in Alzheimer's dementia, but in fact they are upregulated in MCI [15], presumably because of reactive upregulations of the enzyme activity in the unaffected hippocampal cholinergic axons. Previous EEG studies [20–26] have shown a decrease—ranging from 8 to 10.5 Hz (low alpha)—of the alpha frequency power band in MCI subjects, when compared to normal elderly controls [20, 27–30]. However, a recent study has shown an increase—ranging from 10.5 to 13 Hz (high alpha)—of the alpha frequency power band, on the occipital region in MCI subjects, when compared to normal elderly and AD patients [30]. These somewhat contradictory findings may be explained by the possibility that MCI subjects have different patterns of plastic organization during the disease and that the activation (or hypoactivation) of different cerebral areas is based on various degrees of hippocampal atrophy. If this hypothesis is true, then EEG changes of rhythmicity have to occur nonproportionally to the hippocampal atrophy, as previously demonstrated in a study of auditory evoked potentials [31].

In a recent study [32], the results confirm the hypothesis that the relationship between hippocampal volume and EEG rhythmicity is not proportional to the hippocampal atrophy, as revealed by the analyses of both the relative band powers and the individual alpha markers. Such a pattern seems to emerge because, rather than a classification based on clinical parameters, discrete hippocampal volume differences (about 1 cm^3^) are analyzed. Indeed, the group with moderate hippocampal atrophy showed the highest increase in the theta power on frontal regions and of the alpha2 and alpha3 powers on frontal and temporoparietal areas.

Recently, two specific EEG markers, theta/gamma and alpha3/alpha2 frequency ratio, have been reliably associated with the atrophy of amygdalo-hippocampal complex [33], as well as with memory deficits, which are a major risk for the development of AD in MCI subjects [34]. Based on the tertiles values of decreasing AHC volume, three groups of AHC growing atrophy were obtained. AHC atrophy is associated with memory deficits as well as with increase of theta/gamma and alpha3/alpha2 ratio. Moreover, when the amygdalar and hippocampal volumes are separately considered, within AHC, the increase of theta/gamma ratio is best associated with amygdalar atrophy whereas alpha3/alpha2 ratio is best associated with hippocampal atrophy.

The role of cerebrovascular (CV) disease and ischemic brain damage in cognitive decline remains controversial. Although not all patients with mild cognitive impairment due to CV damage develop a clinically defined dementia, all such patients are at risk and could develop dementia in the 5 years following the detection of cognitive decline. Cognitive impairment due to subcortical CV damages is thought to be caused by focal or multifocal lesions involving strategic brain areas. These lesions in basal ganglia, thalamus, or connecting white matter induce interruption of thalamocortical and striatocortical pathways. As a consequence, deafferentation of frontal and limbic cortical structures is produced. The pattern of cognitive impairment is consistent with models of impaired cortical and subcortical neuronal pathways [36]. Even when CV pathology appears to be the main underlying process, the effects of the damaged brain parenchyma are variable and, therefore, the clinical, radiological, and pathological appearances may be heterogeneous. A neurophysiological approach could be helpful in differentiating structural from functional CV damage [35]. The quantitative analysis of electroencephalographic (EEG) rhythms in resting subjects is a low-cost but still powerful approach to the study of elderly subjects in normal aging, MCI, and dementia. The aim of this study was to compare specific EEG markers that could be useful for diagnostic and prognostic purpose in the investigation of patients with cognitive decline.

## 2. Materials and Methods

### 2.1. Subjects 

#### 2.1.1. General Considerations about Recruitment

All the subjects in the study were recruited from the same cohort in the Memory Clinic of the Scientific Institute for Research and Care (IRCCS) of Alzheimer's and psychiatric diseases “Fatebenefratelli” in Brescia, Italy. All experimental protocols had been approved by the local Ethics Committee. Informed consent was obtained from all participants or their caregivers, according to the Code of Ethics of the World Medical Association (Declaration of Helsinki). The difference in the size of the populations (cerebrovascular and degenerative impairment) is due to technical reasons linked to the MRI analysis.

**Cerebrovascular Impairment**

For the present study, 99 subjects with MCI were recruited.  Table 1 shows the main features of this group.

**Degenerative Impairment**

For the present study, 79 subjects with MCI were recruited.  Table 3 shows the main characteristics of the group.

### 2.2. Shared Procedures

#### 2.2.1. EEG Recordings

All recordings were obtained in the morning with subjects resting comfortably. Vigilance was continuously monitored in order to avoid drowsiness.

The EEG activity was recorded continuously from 19 sites by using electrodes set in an elastic cap (Electro-Cap International, Inc.) and positioned according to the 10–20 International system (Fp1, Fp2, F7, F3, Fz, F4, F8, T3, C3, Cz, C4, T4, T5, P3, Pz, P4, T6, O1, and O2). The ground electrode was placed in front of Fz. The left and right mastoids served as reference for all electrodes. The recordings were used off line to rereference the scalp recordings to the common average. Data were recorded with a band-pass filter of 0.3–70 Hz and digitized at a sampling rate of 250 Hz (BrainAmp, BrainProducts, Germany). Electrodes-skin impedance was set below 5 k*Ω*. Horizontal and vertical eye movements were detected by recording the electrooculogram (EOG). The recording lasted 5 minutes, with subjects with closed eyes. Longer recordings would have reduced the variability of the data, but they would also have increased the possibility of slowing of EEG oscillations due to reduced vigilance and arousal. EEG data were then analyzed and fragmented off line in consecutive epochs of 2 seconds, with a frequency resolution of 0.5 Hz. The average number of epochs analyzed was 140 ranging from 130 to 150. The EEG epochs with ocular, muscular, and other types of artifacts were discarded.

#### 2.2.2. Analysis of Individual Frequency Bands. 

A digital FFT-based power spectrum analysis (Welch technique, Hanning windowing function, no phase shift) computed—ranging from 2 to 45 Hz—the power density of EEG rhythms with a 0.5 Hz frequency resolution. Methods are exposed in detail elsewhere [32, 35, 37]. Briefly, two anchor frequencies were selected according to the literature guidelines [38], that is, the theta/alpha transition frequency (TF) and the individual alpha frequency (IAF) peak. Based on TF and IAF, we estimated the following frequency band range for each subject: delta, theta, low alpha band (alpha1 and alpha2), and high alpha band (alpha3). Moreover, individual beta and gamma frequencies were computed. Three frequency peaks were detected in the frequency range from the individual alpha 3 frequency band and 45 Hz. These peaks were named beta 1 peak (IBF 1), beta 2 peak (IBF 2), and gamma peak (IGF). Based on peaks, the frequency ranges were determined. Beta1 ranges from alpha 3 to the lower spectral power value between beta 1 and beta 2 peak; beta2 frequency ranges from beta 1 to the lower spectral power value between beta 2 and gamma peak; gamma frequency ranges from beta 2 to 45 Hz, which is the end of the range considered. The frequency range was determined for each patient. On average, the boundaries of the frequency bands were as follows: delta 2.9–4.9 Hz; theta 4.9–6.9 Hz; alpha1 6.9–8.9 Hz; alpha 2 8.9–10.9 Hz; alpha3 10.9–12-9 Hz; beta1 12,9–19,2 Hz; beta2 19.2–32.4; gamma 32.4–45. In the frequency bands determined in this way, the relative power spectra for each subject were computed. The relative power density for each frequency band was computed as the ratio between the absolute power and the mean power spectra from 2 to 45 Hz. Finally, the theta/gamma and alpha3/alpha2 relative power ratio were computed and analyzed. The analysis of other frequencies was not in the scope of this study.

#### 2.2.3. Diagnostic Criteria

In this study we enrolled subjects afferents to the Scientific Institute of Research and Care Fatebenefratelli in Brescia, Italy. Patients were taken from a prospective project on clinical progression of MCI. The project was aimed to study the natural history of nondemented persons with apparently primary cognitive deficits, not caused by psychic (anxiety, depression, etc.) or physical (uncontrolled heart disease, uncontrolled diabetes, etc.) conditions. Patients were rated with a series of standardized diagnostic tests, including the Mini-Mental State Examination (MMSE; [39]), the Clinical Dementia Rating Scale (CDRS; [40]), the Hachinski Ischemic Scale (HIS; [41]), and the Instrumental and Basic Activities of Daily Living (IADL, BADL, [42]). In addition, patients were subjected to diagnostic neuroimaging procedures (magnetic resonance imaging (MRI)) and laboratory blood analysis to rule out other causes of cognitive impairment. 

The present inclusion and exclusion criteria for MCI were based on previous seminal studies [2, 3, 43–46]. Inclusion criteria in the study were all of the following: (i) complaint by the patient or report by a relative or the general practitioner of memory or other cognitive disturbances; (ii) Mini-Mental State Examination (MMSE; [39]) score of 24 to 27/30 or MMSE of 28 and higher plus low performance (score of 2/6 or higher) on the clock drawing test [47]; (iii) sparing of instrumental and basic activities of daily living or functional impairment stably due to causes other than cognitive impairment, such as physical impairments, sensory loss, and gait or balance disturbances. Exclusion criteria were any one of the following: (i) age of 90 years and older; (ii) history of depression or psychosis of juvenile onset; (iii) history or neurological signs of major stroke; (iv) other psychiatric diseases, epilepsy, drug addiction, alcohol dependence; (v) use of psychoactive drugs including acetylcholinesterase inhibitors or other drugs enhancing brain cognitive functions; (vi) current or previous uncontrolled or complicated systemic diseases (including diabetes mellitus) or traumatic brain injuries.

All patients underwent (i) semistructured interview with the patient and—whenever possible—with another informant (usually the patient's spouse or a child) by a geriatrician or neurologist; (ii) physical and neurological examinations; (iii) performance-based tests of physical function, gait, and balance; (iv) neuropsychological assessment evaluating verbal and nonverbal memory, attention, and executive functions (Trail Making Test B-A; Clock Drawing Test; [47, 48]), abstract thinking (Raven matrices; [49]), frontal functions (Inverted Motor Learning; [50]), language (Phonological and Semantic fluency; Token test; [51]), and apraxia and visuoconstructional abilities (Rey figure copy; [52]); (v) assessment of depressive symptoms with the Center for Epidemiologic Studies Depression Scale (CES-D; [53]). Given the aim of the study to evaluate the impact of vascular damage on EEG rhythms, in this study we did not consider the clinical subtype of MCI, that is, amnesic, nonamnesic, or multiple domain.

#### 2.2.4. Magnetic Resonance Imaging (MRI) and CV Damage Evaluation

Magnetic resonance (MR) images were acquired using a 1.0 Tesla Philips Gyroscan. Axial T2 weighted, proton density (DP), and fluid attenuated inversion recovery (FLAIR) images were acquired with the following acquisition parameters: TR = 2000 ms, TE = 8.8/110 ms, flip angle = 90°, field of view = 230 mm, acquisition matrix 256 × 256, slice thickness 5 mm for T2/DP sequences and TR = 5000 ms, TE = 100 ms, flip angle = 90°, field of view = 230 mm, acquisition matrix 256 × 256, slice thickness 5 mm for FLAIR images. 

Subcortical cerebrovascular disease (sCVD) was assessed using the rating scale for age-related white matter change (ARWMC) on T2-weighted and FLAIR MR images. White matter changes (WMC) was rated by a single observer (R.R.) in the right and left hemispheres separately in frontal, parietooccipital, temporal, and infratentorial areas and basal ganglia on a 4-point scale. The observer of white matter changes was blind to the clinical information of the subjects. Subscores of 0, 1, 2, and 3 were assigned in frontal, parieto-occipital, temporal, and infratentorial areas for no WMC, focal lesions, beginning confluence of lesions, and diffuse involvement of the entire region, respectively. Subscores of 0, 1, 2, and 3 were assigned in basal ganglia for no WMC, 1 focal lesion, more than 1 focal lesion, and confluent lesions, respectively. Total score was the sum of subscores for each area in the left and right hemisphere, ranging from 0 to 30. As regards the ARWMC scale, the interrater reliability, as calculated with weighted *k* value, was 0.67, indicative of moderate agreement [54]. We assessed test-retest reliability on a random sample of 20 subjects. The intraclass correlation coefficient was 0.98, values above 0.80 being considered indicative of good agreement.

Based on increasing subcortical CV damage, the 99 MCI subjects were subsequently divided in 4 subgroups along the range between the minimum and maximum ARWMC score (resp. 0 and 15). In order to have the higher sensibility to the CV damage, the first group was composed by subjects with score = 0. The other groups were composed according to equal range ARWMC scores. As a consequence, we obtained the following groups: group 1 (G1): no vascular damage, CV score 0; group 2 (G2): mild vascular damage, CV score 1–5; group 3 (G3): moderate vascular damage, CV score 6–10; group 4 (G4): severe vascular damage, CV score 11–15. 

Table 1 reports the mean values of demographic and clinical characteristics of the 4 subgroups. 

#### 2.2.5. Statistical Analysis

Preliminarily, any significant difference between groups in demographic variables, age, education, and gender as well as MMSE score was taken into account. Only education showed a significant difference between groups (*P* < 0.03). For avoiding confounding effect, subsequent statistical analyses of variance (ANOVA) were carried out using age, education, gender, and MMSE score as covariates. Duncan's test was used for post hoc comparisons. For all statistical tests the significance was set to *P* < 0.05.

A second session of ANOVA was performed on EEG relative power data. In this analysis, group factor was the independent variable and frequency band power (delta, theta, alpha1, alpha2, and alpha 3) the dependent variable. 

As successive step, to evaluate the presence of EEG indexes that correlate specifically with vascular damage, we performed statistical analyses to evaluate the specificity of the following ratios: theta/alpha1, using as covariate also TF; alpha2/alpha3 using as covariate also IAF and alpha1/alpha2 with both TF and IAF as covariate. Moreover, we performed correlations (Pearson's moment correlation) between CV damage score and frequency markers (TF and IAF), spectral power, and MMSE. Finally, we performed a control statistical analysis with 4 frequency bands, considering alpha1 and alpha2 as single band (low alpha). This analysis had the aim of verifying if the low alpha, when considered as a whole, has the same behavior.

## 3. Results

### 3.1. Vascular MCI

**Figure 1:**
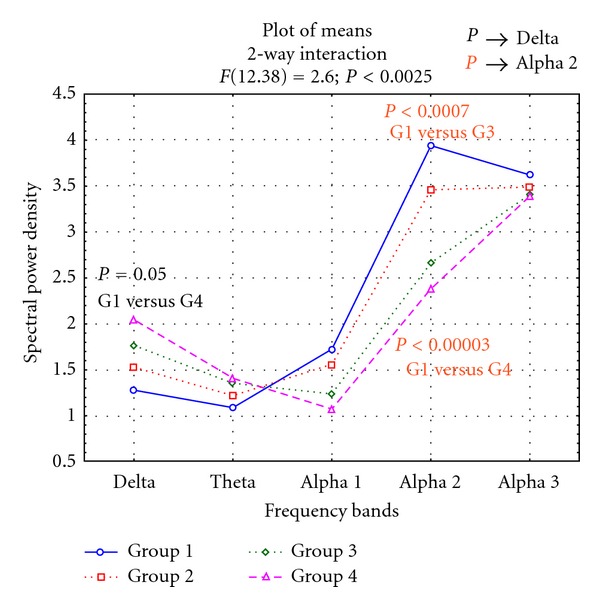
Statistical ANOVA interaction among groups, factor and relative band power (delta, theta, alpha1, alpha2, and alpha3). In the diagram the difference in delta and alpha2 power among groups is also indicated, based on Duncan's post hoc testing. G1, group 1: no vascular damage; G2, group 2: mild vascular damage; G3, group 3: moderate vascular damage; G4, group 4: severe vascular damage [32, 35].

Figure 1 displays the results for ANOVA analysis of these data showing a significant interaction between group and Band factors (*F*(12.380) = 2.60; *P* < 0.002). Interestingly, Duncan's post hoc testing showed a significant higher delta power in G4 compared to G1 (*P* < 0.050) and a significant higher alpha2 power in G1 compared to G3 and G4 (*P* < 0.000). On the contrary, no differences were found in theta, alpha1, and alpha 3 band powers. Moreover, a closer look at the data, in respect to the alpha1 frequency, showed a decrease proportional to the degree of CV damage very similar to alpha2 band, although not significant. On the contrary, in the alpha3 band power this trend was not present, suggesting that vascular damage had no impact on this frequency band.

The correlation analysis between CV score and spectral band power showed a significant positive correlation with delta power (*r* = 0.221; *P* < 0.03) a significant negative correlation with alpha1 (*r* = −0.312; *P* < 0.002) and alpha2 power (*r*= − 0.363; *P* < 0.0003). The correlations between CV score with theta power (*r* = 0.183; *P* = 0.07) and alpha3 power (*r* = −0.002; *P* = 0.93) were not significant as well as the correlation between CV score and MMSE (*r* = −0.07; *P* = 0.4).

Table 2 displays the values of the theta/alpha1 and alpha2/alpha3 power ratio. The statistical analysis of the theta/alpha1 ratio showed a main effect of group(*F*(3.91) = 15.51; *P* < 0.000). Duncan's post hoc testing showed a significant increase of the theta/alpha1 ratio between G1 and G2 in respect to G3 and G4 (*P* < 0.000). Moreover, the increase of this ratio was significant also between G3 and G4 (*P* < 0.04). The statistical analysis of the alpha2/alpha3 power ratio showed a main effect of group(*F*(3.91) = 4.60; *P* < 0.005). Duncan's post hoc testing showed a significant decrease of the ratio between G1 and G3 (*P* < 0.02), G1 and G4 (*P* < 0.010), and G2 and G4 (*P* < 0.05). The statistical analysis of the alpha1/alpha2 ratio did not show the main effect of group(*P* < 0.2).

### 3.2. MRI Scans and Amygdalohippocampal Atrophy Evaluation

MRI scans were acquired with a 1.0 Tesla Philips Gyroscan at the Neuroradiology Unit of the Città di Brescia Hospital, Brescia. The following sequences were used to measure hippocampal and amygdalar volumes: a high-resolution gradient echo T1-weighted sagittal 3D sequence (TR = 20 ms, TE = 5 ms, flip angle = 30°, field of view = 220 mm, acquisition matrix = 256 × 256, and slice thickness = 1.3 mm) and a fluid-attenuated inversion recovery (FLAIR) sequence (TR = 5000 ms, TE = 100 ms, flip angle = 90°, field of view = 230 mm, acquisition matrix = 256 × 256, and slice thickness = 5 mm). Hippocampal, amygdalar, and white matter hyperintensities (WMHs) volumes were obtained for each subject. The hippocampal and amygdalar boundaries were manually traced on each hemisphere by a single tracer with the software program DISPLAY (McGill University, Montreal, Canada) on contiguous 1.5 mm slices in the coronal plane. The amygdala is an olive-shaped mass of gray matter located in the superomedial part of the temporal lobe, partly superior and anterior to the hippocampus. The starting point for amygdala tracing was at the level where it is separated from the entorhinal cortex by intrarhinal sulcus, or tentorial indentation, which forms a marked indent at the site of the inferior border of the amygdala. The uncinate fasciculus, at the level of basolateral nuclei groups, was considered as the anterior-lateral border. The amygdalo-striatal transition area, which is located between lateral amygdaloid nucleus and ventral putamen, was considered as the posterior-lateral border. The posterior end of amygdaloid nucleus was defined as the point where gray matter starts to appear superior to the alveolus and lateral to the hippocampus. If the alveolus was not visible, the inferior horn of the lateral ventricle was employed as border [33]. The starting point for hippocampus tracing was defined as the hippocampal head when it first appears below the amygdala, the alveus defining the superior and anterior border of the hippocampus. The fimbria was included in the hippocampal body, while the grey matter rostral to the fimbria was excluded. The hippocampal tail was traced until it was visible as an oval shape located caudally and medially to the trigone of the lateral ventricles [32, 35]. The intraclass correlation coefficients were 0.95 for the hippocampus and 0.83 for the amygdala.

White matter hyperintensities (WMHs) were automatically segmented on the FLAIR sequences by using previously described algorithms [32, 35]. Briefly, the procedure includes (i) filtering of FLAIR images to exclude radiofrequency inhomogeneities, (ii) segmentation of brain tissue from cerebrospinal fluid, (iii) modelling of brain intensity histogram as a Gaussian distribution, and (iv) classification of the voxels whose intensities were ≥3.5 SDs above the mean as WMHs [32, 35], Total WMHs volume was computed by counting the number of voxels segmented as WMHs and multiplying by the voxel size (5 mm^3^). To correct for individual differences in head size, hippocampal, amygdalar and WMHs volumes were normalized to the total intracranial volume (TIV), obtained by manually tracing with DISPLAY the entire intracranial cavity on 7 mm thick coronal slices of the T1 weighted images. Both manual and automated methods used here have advantages and disadvantages. Manual segmentation of the hippocampus and amygdala is currently considered the gold standard technique for the measurement of such complex structures. The main disadvantages of manual tracing are that it is operator dependent and time consuming. Conversely, automated techniques are more reliable and less time-consuming but may be less accurate when dealing with structures without clearly identifiable borders. This, however, is not the case for WMHs, which appear as hyperintense on FLAIR sequences. 

Left and right hippocampal as well as amygdalar volumes were estimated and summed to obtain a total volume (individual) of both anatomical structures. In turn, total amygdalar and hippocampal volumes were summed obtaining the whole AHC volume. AHC (whole) volume has been divided in tertiles obtaining three groups. In each group hippocampal and amygdalar volumes (within AHC) have been computed. The last volumes were compared with the previous obtained individual (hippocampal and amygdalar) volumes.

### 3.3. Statistical Analysis and Data Management

The analysis of variance (ANOVA) has been applied as statistical tool. At first, any significant differences among groups in demographic variables, that is, age, education, MMSE score, and morphostructural characteristics, that is, AHC, hippocampal, amygdalar, and white matter hyperintensities (WMHs) volume, were evaluated (Table 1). Greenhouse-Geisser correction and Mauchly's sphericity test were applied to all ANOVAs. In order to avoid a confounding effect, ANOVAs were carried out using age, education, MMSE score, and WMHs as covariates. For all statistical tests the significance level was set at *P* < 0.05. Duncan's test was used for post hoc comparisons. In a first study [32] we considered only the hippocampal volume and obtained 4 subgroups based on the hippocampal volume atrophy. In a subsequent study [33], given the importance of the amigdala in both degenerative pathology and brain rhythm generation, we considered the amygdalo-hippocampal volume as a whole, in order to investigate the entire AHC and to focus on the hippocampal volume within the AHC itself. In this case we obtain three groups based on AHC atrophy. Both the first and the second studies were performed on the same 79 subjects. The difference in groups subdivision (4 versus 3) was mandatory to obtain group with volumetric statistical significant difference. 

At first, we choose to focus on the changes of brain rhythmicity induced from hippocampal atrophy alone. Subjects were subdivided in four groups based on hippocampal volume of a normal control sample matched for age, sex, and education as compared to the whole MCI group. In the normal group the female/male ratio was 93/46, mean age was 68.9 (SD ± 10.3), mean education was years 8.9 (SD ± 9.4). The mean and standard deviation of the hippocampal volume in the normal old population of 139 subjects were 5.72 ± 1.1 cm^3^. In this way, 4 groups were obtained: the no atrophy group with hippocampal volume equal or superior to the normal mean (total hippocampal volume from 6.79 cm^3^ to 5.75 cm^3^; G1); the mild atrophy group which has hippocampal volume within 1.5 SD below the mean of hippocampal normal control value (total hippocampal volume from 5.70 to 4.70 cm^3^; G2); the moderate atrophy group which has hippocampal volume between 1.5 and 3 SD below the mean of normal hippocampus (total hippocampal volume from 4.65 to 3.5 cm^3^; G3); the severe atrophy group which has hippocampal volume between 3 and 4.5 SD below the mean of hippocampal normal control volume (total hippocampal volume from 3.4 to 2.53 cm^3^; G4). The rationale for the selection of 1,5 SD was to obtain reasonably pathological groups based on hippocampal volume. A SD below 1.5 could recollect still normal population based on hippocampal volume. On the other side, a SD over 1.5 could not allow an adequate size of all subgroups in study.

Subsequently, ANOVA was performed in order to verify (1) the difference of AHC volume among groups; (2) the difference of hippocampal and amygdalar volume within AHC among groups; (3) the difference of hippocampal and amygdalar volume individually considered among groups; (4) NPS impairment based on ACH atrophy.

Moreover, as a control analysis, in order to detect if difference in EEG markers was linked to significant difference in volume measurements, the volume of hippocampus within AHC was compared with the hippocampal volume individually considered, and the amygdalar volume within AHC was compared with the amygdalar volume individually considered. This control analysis was performed through a paired *t*-test.

Subsequently, ANOVA was performed in order to check differences in theta/gamma and alpha3/alpha2 relative power ratio in the three groups ordered by decreasing tertile values of the whole AHC volume. In each ANOVA, group was the independent variable, the frequency ratios was the dependent variable, and age, education, MMSE score, and WMHs were used as covariates. Duncan's test was used for post-hoc comparisons. For all statistical tests the significance level was set at *P* < 0.05. 

In order to check closer association with EEG markers, hippocampal volume and amygdalar volume within AHC were analyzed separately. A control analysis was carried out also on the individual hippocampal and amygdalar volumes based on decreasing tertile values for homogeneity with the main analysis.

### 3.4. Amigdalohyppocampal Atrophy MCI

**Figure 2:**
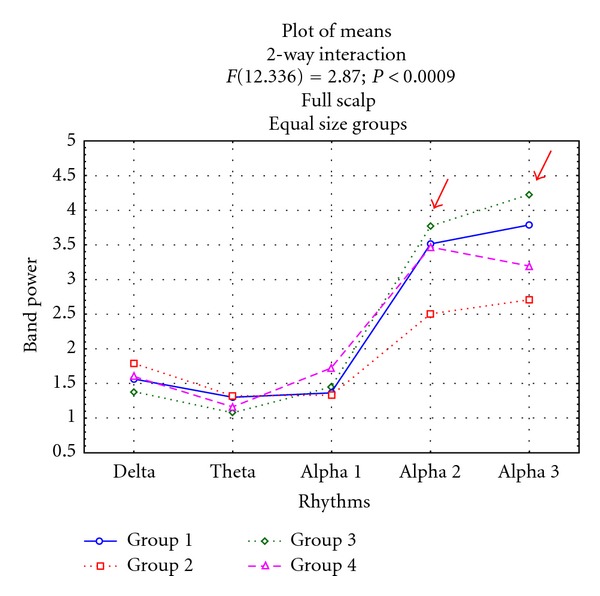
Statistical ANOVA interaction among group factors and relative band powers (delta, theta, alpha1, alpha2, and alpha3), on the full scalp region. The groups are based on mean and standard deviations in a normal elderly sample. Group 1: no hippocampal atrophy; group 2: mild hippocampal atrophy; group 3: moderate hippocampal atrophy; group 4: severe hippocampal atrophy. Post hoc results are indicated in the diagram (see [32, 35]).

Figure 2 displays the results for ANOVA analysis performed on 4 groups of MCI considering growing values of hippocampal atrphy. The results show a significant interaction between group and band power (*F*(12,336) = 2,36); *P* < 0.007). Duncan's post hoc testing showed that G3 group has the highest alpha2 and alpha3 power statistically significant with respect to all other groups (*P* < 0.05; *P* < 0.006, resp.). The same trend was present in the subsidiary ANOVA. These results show that the relationship between hippocampal atrophy and EEG relative power is not proportional to the hippocampal atrophy and highlight that the group with a moderate hippocampal volume had a particular pattern of EEG activity as compared to all other groups.

Table 3 summarizes the ANOVA results of demographic variables, that is, age, education, MMSE score, and morphostructural characteristics, that is, hippocampal, amygdalar, and white matter hyperintensities volume in the whole MCI cohort as well as in the three subgroups in study. Hippocampal and aymgdalar volumes are considered as parts of the whole AHC volume and are individually considered. Significant statistical results were found in hippocampal and amygdalar volume both within the AHC (resp., *F*_2,76_ = 92.74; *P* < 0.00001  and  *F*_2,76_ = 33.82; *P* < 0.00001) and were individually considered (resp., *F*_2,76_ = 157.27; *P* < 0.00001  and  *F*_2,76_ = 132.5; *P* < 0.00001). The global AHC volume also showed significant results (*F*_2,76_ = 159.27; *P* < 0.00001). Duncan's post hoc test showed a significant increase (*P* < 0.01) in all comparisons. The paired *t*-test showed significant difference between the volume of ACH-amygdala and amygdalar volume individually considered in the first group (*P* < 0.03). The amygdalar volume difference in the other groups (resp. *P* = 0.2 and 0.1) as well as the difference in the volume of AHC-hippocampus versus individual hippocampus (*P* = 0.4 in the first, *P* = 0.5 in the second, and *P* = 0.1 in the third group) was not statistically significant.

Tables 3 and 4 show the results of theta/gamma and alpha3/alpha2 ratio in the groups based on the decrease of whole AHC volume as well as, within the same group, the decrease of hippocampal and amygdalar volumes separately considered. ANOVA shows results towards significance when amygdaloippocampal volume is considered globally in both theta/gamma (*F*_2,76_ = 2.77; *P* < 0.06) and alpha3/alpha2 ratio (*F*_2,76_ = 2.71; *P* < 0.07). When amygdalar and hippocampal volumes were considered separately, ANOVA results revealed significant main effect of Group, respectively, in theta/gamma ratio analysis (*F*_2,76_ = 3.46; *P* < 0.03) for amygdalar and alpha3/alpha2 ratio for hippocampal (*F*_2,76_ = 3.38; *P* < 0.03) decreasing volume. The ANOVA did not show significant results in theta/gamma ratio when considering hippocampal volume (*F*_2,76_ = 0.3; *P* < 0.7) and in alpha3/alpha2 ratio when considering amygdalar volume (*F*_2,76_ = 1.46; *P* < 0.2). The control analysis (individual volumes) did not show any significant result neither for hippocampal (theta/gamma, *F*_2,76_ = 0.3; *P* < 0.7; alpha3/alpha2, *F*_2,76_ = 2.15; *P* < 0.1) nor for amygdalar volume (theta/gamma, *F*_2,76_ = 0.76; *P* < 0.4; alpha3/alpha2, *F*_2,76_ = 2.15; *P* < 0.1).

## 4. Discussion

**MCI and EEG Markers: Degenerative versus Vascular Impairment**

A large body of literature has previously demonstrated that in subjects with cognitive decline an increase of theta relative power [32, 33, 35], a decrease of gamma relative power [32, 33, 35, 55], and an increase of high alpha as compared to low alpha band are present [33]. On the whole theta/gamma ratio and alpha3/alpha2 ratio could be considered reliable EEG markers of cognitive decline.

The amygdalohippocampal network is a key structure in the generation of theta rhythm. More specifically, theta synchronization is increased between LA and CA1 regions of hippocampus during long-term memory retrieval, but not during short-term or remote memory retrieval [56, 57]. Theta synchronization in AHC appears to be apt to improve the neural communication during memory retrieval [57]. On the other hand, the retrieval of hippocampus-dipendent memory is provided by the integrity of CA3-CA1 interplay coordinated by gamma oscillations [58]. Our results confirm and extend all previous findings. The atrophy of AHC determines increasing memory deficits. The brain oscillatory activity of this MCI state is characterized by an increase of theta/gamma ratio and alpha3/alpha2 relative power ratio, confirming the overall reliability of these EEG markers in cognitive decline. Our results suggest that theta synchronization is mainly due to the amygdala activation or as a subsequent final net effect within the AHC functioning driven by the amygdala excitation. The increase in theta activities in AHC, representing an increase in neuronal communication apt to promote or stabilize synaptic plasticity in relation to the effort to retention of associative memories [59], could be active also during an ongoing degenerative process. The excitation mechanism could be facilitated by the loss of GABA inhibitory process, determining the decrease of gamma rhythm generation [58, 60].

As regards the CV damage, our results showed that the CV damage affected both delta and low alpha band power (alpha1 and alpha2). In the delta band we observed a power increase proportional to the CV damage, with a significant increase in the group with severe CV damage, as compared to the no-CV-damage group. The impact of the CV damage on the delta power was confirmed by the significant positive correlation between CV damage score and delta power itself.

The increase in the delta band power could be explained as a progressive cortical disconnection due to the slowing of the conduction along corticosubcortical connecting pathways.

This result confirmed the increase in the delta band power we had observed in CV patients, as compared to normal elderly subjects [37]. It is to be noted that the increase in the delta band power reflects a global state of cortical deafferentation, due to various anatomofunctional substrates, such as stages of sleep, metabolic encephalopathy, or corticothalamocortical dysrhythmia [61]. In the low alpha band power, we observed a significant decrease in the alpha 2 band power for the groups with moderate and severe CV damage, as compared to the no-CV-damage group. In the alpah1 frequency band, there was a similar decrease although it did not reach statistically significant values. These results were confirmed by a correlation analysis that showed a significant negative correlation between CV damage score and alpha1 and alpha2 band powers. In our results, the CV damage did not show any impact on the alpha3 (or high alpha) power. This is a confirmation of what we found in the previous study, where no differences between VaD patients and normal elderly (but not in AD versus normal elderly) subjects were detected in the alpha3 power.

Together, these results could suggest different generators for low alpha and high alpha frequency bands. In particular, the low alpha band power could affect corticosubcortical mechanisms, such as corticothalamic, corticostriatal, and corticobasal ones. This could explain the sensitivity of the low alpha frequency band to subcortical vascular damage. On the contrary, the alpha3 band power could affect to a greater extent those corticocortical interactions based on synaptic efficiency prone to degenerative rather than CV damages [38, 62]. In order to find reliable indices of CV damage, we checked the theta/alpha1 band power ratio. Previous studies have shown the reliability of this kind of approach in quantitative EEG in demented patients [21]. The importance of this ratio lies in the presence of such frequency bands on the opposite side of the TF, that is, the EEG frequency index most significantly affected by the CV damage. So, the theta/alpha1 band power ratio could represent the most sensitive EEG marker of CV damage. The results showed a significant increase of the theta/alpha1 band power ratio in moderate and severe CV damage groups, as compared to mild and no-CV-damage groups. This ratio increase establishes a proportional increase of the theta band power relative to the alpha 1 band power with respect to the CV damage, even though a significant increase in the theta band power per se (or a decrease in the alpha 1 band power per se) is not present. This could suggest a reliable specificity for the theta/alpha 1 band power ratio in focusing the presence of a subcortical CV damage.

**MCI, Cognitive Deficits (Memory and Attention), and EEG Activity**

The vulnerability and damage of the connections of hippocampus with amygdala could affect reconsolidation of long-term memory and give rise to memory deficits and behavioural symptoms. Several experiments show that amygdala activity is prominent during the period of intense arousal, for example, the anticipation of a noxious stimulus [63] or the maintenance of vigilance to negative stimuli [64]. So far, the theta synchronization induced by the amygdala is deeply involved in endogenous attentional mechanism. Interestingly, the increase of high alpha synchronization has been found in internally cued mechanisms of attention, associated with inhibitory top-down processes [62]. Of note, the amygdala is intimately involved in the anatomophysiological anterior pathways of attention through its connections with anterior cingulated cortex, anteroventral, anteromedial, and pulvinar thalamic nuclei [65]. The particular role of amygdala in negative human emotions could indicate that AHC atrophy is associated with excessive level of subcortical inputs not adequately filtered by attentive processing, determining fear and anxiety and generating cognitive interference in memory performance. Of note, an altered emotional response is very frequent in MCI patients [66, 67]. In a feedback process, this alteration could determine a general state of “hyperattention” during which top-down internal processes prevail on the bottom-up phase, altering attention mechanism and preventing a correct processing of sensory stimuli. Focused attention has been found impaired in MCI patients in particular when they have to benefit from a cue stimulus [68–72]. This particular state could be useful for maintaining a relatively spared global cognitive performance, whereas it could fail when a detailed analysis of a sensory stimulus is required. This “hyperattentive” state could represent the attempt to recollect memory and/or spatial traces from hippocampus and to combine them within associative areas connected with hippocampus itself.

The increase of alpha3/alpha2 ratio in our results supports the concomitance of anterior attentive mechanism impairment in subjects with MCI, even though there are not overt clinical deficits. The major association of the increase of alpha3/alpha2 ratio with the hippocampal formation within the AHC suggests that this filter activity is carried out by hippocampus and its input-output connections along anterior attentive circuit and AHC. Interestingly, a recent work has demonstrated that the mossy fiber (MF) pathway of the hippocampus connects the dentate gyrus to the auto-associative CA3 network and the information it carries is controlled by a feedforward circuit combining disynaptic inhibition with monosynaptic excitation. Analysis of the MF-associated circuit revealed that this circuit could act as a highpass filter [73].

As regards CV impaired subjects, the natural history of a group of subjects at very high risk for developing dementia due to subcortical vascular damage (subcortical vascular MCI (svMCI)) has recently been described [74, 75]. In such study, MCI patients with CV etiology developed a distinctive clinical phenotype characterized by poor performance on frontal tests and neurological features of parkinsonism without tremor (impairment of balance and gait). These clinical features could be explained by our results. In CV patients, we observed a slowing of the a frequency in the two groups with greater CV damage, as compared to the groups with lesser CV damage. This is in line with a previous study [37] showing the major effect of the CV damage, in patients with vascular dementia (VaD) versus normal elderly and Alzheimer's patients. A reasonable (although speculative) explanation of the present results is that the CV-damage-induced slowing of the a frequency start point could be mainly attributed to the lowering of the conduction time of synaptic action potentials throughout corticosubcortical fibers, such as corticobasal or corticothalamic pathways [76]. In fact, experimental models have previously shown that the EEG frequency is due to axonal delay and synaptic time of corticosubcortical interactions [77–79]. Most interestingly, other studies have demonstrated that fiber myelination affects the speed propagation along cortical fibers and that this parameter is strictly correlated to the frequency range recorded on the scalp. In fact, a theoretical model considering a mean speed propagation in white matter fibers of 7.5 m/s (together with other parameters) is associated with a fundamental mode frequency of 9 Hz [80], that is, the typical mode of scalp-recorded EEG. It is to be noted that a correlation between white matter damage and widespread slowing of EEG rhythmicity was found in other studies, following the presence of cognitive decline [81], multiple sclerosis [82], or cerebral tumors [83]. The increase of the theta/alpha1 band power ratio in moderate and severe CV damage groups, as compared to mild and no-CV-damage groups, could be the neurophysiological correlate of the functional slowing of the speed propagation of the electric signals in vascular impaired subjects.

## Figures and Tables

**Table 1:** Mean values ± standard error of demographic characteristics, neuropsychological and ARWMC scores of the MCI subgroups. F/M: female/male. Age and education are expressed in years. Group 1: no vascular damage; group 2: mild vascular damage; group 3: moderate vascular damage; group 4: severe vascular damage.

	Group 1	Group 2	Group 3	Group 4
Subjects (f/m)	27 (18/9)	41 (31/10)	19 (10/9)	12 (9/3)
Age	70.1 (±1.7)	69.9 (±1.1)	69.7 (±1.9)	70.5 (±2.4)
Education	7.1 (±0.7)	7 (±0.6)	7 (±0.9)	10 (±1.6)
MMSE	26.7 (±0.4)	26.5 (±0.4)	27 (±0.4)	26.1 (±0.7)
ARWMC scale	0	1–5	6–10	11–15

**Table 2:** Mean values ± standard error of theta/alpha1, alpha1/alpha2, and alpha2/alpha3 ratios in the MCI subgroups. Group 1: no vascular damage; group 2: mild vascular damage; group 3: moderate vascular damage; group 4: severe vascular damage.

	Group 1	Group 2	Group 3	Group 4
*θ*/*α*1	0.7 (±0.05)	0.77 (±0.05)	1.17 (±0.05)	1.39 (±0.14)
*α*1/*α*2	0.46 (±0.03)	0.5 (±0.03)	0.53 (±0.05)	0.47 (±0.04)
*α*2/*α*3	1.27 (±0.12)	1.16 (±0.1)	0.85 (±0.05)	0.79 (±0.07)

**Table 3:** Mean values ± standard deviation of sociodemographic characteristics, MMSE scores, white matter hyperintensities, and hippocampal and amygdalar volume measurements. Hippocampal and amygdalar volumes refer tothe whole amygdalohippocampal complex (AHC) and are singularly considered (individual). The *t*-test refers to AHC versus individual volume in each group.

	MCI cohort	Group 1	Group 2	Group 3	*P* value (ANOVA)
Number of subjects (f/m)	79 (42/37)	27 (14/13)	27 (15/12)	25 (13/12)	
Age (years)	69.2 ± 2.3	66.8 ± 6.8	69.4 ± 8.7	71.5 ± 6.9	0.1
Education (years)	7.7 ± 0.8	8.3 ± 4.5	6.7 ± 3.1	8.2 ± 4.6	0.2
MMSE	27.1 ± 0.4	27.5 ± 1.5	27.4 ± 1.5	26.6 ± 1.8	0.1
Total AHC volume	6965.3 ± 1248.8	8151.2 ± 436.4	7082.7 ± 266.9	5661.8 ± 720.4	0.00001
AHC-hippocampal volume (mm^3^)	4891.7 ± 902.6	5771.6 ± 361.1	4935.6 ± 380.9	3967.9 ± 650.3	0.00001
AHC-amygdalar volume (mm^3^)	2073.5 ± 348.7	2379.6 ± 321.3	2147.1 ± 301.3	1693.9 ± 288.5	0.0001
Individual hippocampal volume (mm^3^)	4889.8 ± 962.4	5809.6 ± 314.2	4969.4 ± 257.6	3890.1 ± 551.4	0.00001
Individual amygdalar volume (mm^3^)	2071.7 ± 446.4	2514.4 ± 259.5	2079.2 ± 122.8	1621.6 ± 185.2	0.0001
White matter hyperintensities (mm^3^)	3.8 ± 0.5	3.2 ± 2.8	4.2 ± 3.8	4.1 ± 3.6	0.7

**Table 4:** Relative power band ratios in amygdalo-hippocampal complex (AHC), hippocampal and amygdalar atrophy. Hippocampal and amygdalar volumes refer to the whole amygdalo-hippocampal complex (AHC) and are singularly considered (individual).

Hippocampal + amygdalar volume	theta/gamma ratio (*μ*v^2^)	*P* value	alpha3/alpha2 ratio (*μ*v^2^)	*P* value
Group1	1.40 ± 0.35	0.06	1.05 ± 0.11	0.07
Group2	1.43 ± 0.35		1.11 ± 0.14	
Group3	1.47 ± 0.44		1.12 ± 0.16	
AHC-hippocampal volume				
Group1	1.39 ± 0.27	0.7	1.04 ± 0.11	0.03
Group2	1.48 ± 0.45		1.11 ± 0.15	
Group3	1.43 ± 0.41		1.12 ± 0.14	
AHC-amygdalar volume				
Group1	1.36 ± 0.37	0.03	1.04 ± 0.13	0.2
Group2	1.44 ± 0.36		1.12 ± 0.16	
Group3	1.49 ± 0.39		1.09 ± 0.11	
Individual hippocampal volume				
Group1	1.39 ± 0.27	0.7	1.04 ± 0.11	0.1
Group2	1.48 ± 0.45		1.07 ± 0.15	
Group3	1.43 ± 0.40		1.10 ± 0.14	
Individual amygdalar volume				
Group1	1.39 ± 0.37	0.1	1.04 ± 0.13	0.4
Group2	1.43 ± 0.36		1.12 ± 0.16	
Group3	1.46 ± 0.39		1.09 ± 0.11	

## References

[1] Flicker C, Ferris SH, Reisberg B (1991). Mild cognitive impairment in the elderly: predictors of dementia. *Neurology*.

[2] Petersen RC, Smith GE, Ivnik RJ (1995). Apolipoprotein E status as a predictor of the development of Alzheimer’s disease in memory-impaired individuals. *Journal of the American Medical Association*.

[3] Petersen RC, Doody R, Kurz A (2001). Current concepts in mild cognitive impairment. *Archives of Neurology*.

[4] Arnold SE, Hyman BT, Flory J, Damasio AR, Van Hoesen GW (1991). The topographical and neuroanatomical distribution of neurofibrillary tangles and neuritic plaques in the cerebral cortex of patients with alzheimer’s disease. *Cerebral Cortex*.

[5] Bobinski M, Wegiel J, Wisniewski HM (1995). Atrophy of Hippocampal formation subdivisions correlates with stage and duration of Alzheimer disease. *Dementia*.

[6] Price JL, Morris JC (1999). Tangles and plaques in nondemented aging and 'preclinical' alzheimer's disease. *Annals of Neurology*.

[7] Schönheit B, Zarski R, Ohm TG (2004). Spatial and temporal relationships between plaques and tangles in Alzheimer-pathology. *Neurobiology of Aging*.

[8] Bennett DA, Schneider JA, Bienias JL, Evans DA, Wilson RS (2005). Mild cognitive impairment is related to Alzheimer disease pathology and cerebral infarctions. *Neurology*.

[9] Frisoni GB, Prestia A, Rasser PE, Bonetti M, Thompson PM (2009). In vivo mapping of incremental cortical atrophy from incipient to overt Alzheimer’s disease. *Journal of Neurology*.

[10] Callen DJA, Black SE, Gao F, Caldwell CB, Szalai JP (2001). Beyond the hippocampus: MRI volumetry confirms widespread limbic atrophy in AD. *Neurology*.

[11] Du AT, Schuff N, Amend D (2001). Magnetic resonance imaging of the entorhinal cortex and hippocampus in mild cognitive impairment and Alzheimer’s disease. *Journal of Neurology Neurosurgery and Psychiatry*.

[12] Gold G, Bouras C, Kövari E (2000). Clinical validity of Braak neuropathological staging in the oldest-old. *Acta Neuropathologica*.

[13] Della-Maggiore V, Chau W, Peres-Neto PR, McIntosh AR (2002). An empirical comparison of SPM preprocessing parameters to the analysis of fMRI data. *NeuroImage*.

[14] Hämäläinen A, Pihlajamäki M, Tanila H (2007). Increased fMRI responses during encoding in mild cognitive impairment. *Neurobiology of Aging*.

[15] Lavenex P, Amaral DG (2000). Hippocampal-neocortical interaction: a hierarchy of associativity. *Hippocampus*.

[16] Dickerson BC, Salat DH, Bates JF (2004). Medial temporal lobe function and structure in mild cognitive impairment. *Annals of Neurology*.

[17] Dickerson BC, Salat DH, Greve DN (2005). Increased hippocampal activation in mild cognitive impairment compared to normal aging and AD. *Neurology*.

[18] Pariente J, Cole S, Henson R (2005). Alzheimer’s patients engage an alternative network during a memory task. *Annals of Neurology*.

[19] Machulda MM, Ward HA, Borowski B (2003). Comparison of memory fMRI response among normal, MCI, and Alzheimer’s patients. *Neurology*.

[20] Jelic V, Johansson SE, Almkvist O (2000). Quantitative electroencephalography in mild cognitive impairment: longitudinal changes and possible prediction of Alzheimer’s disease. *Neurobiology of Aging*.

[21] Jelic V, Julin P, Shigeta M (1997). Apolipoprotein E *ε*4 allele decreases functional connectivity in Alzheimer’s disease as measured by EEG coherence. *Journal of Neurology Neurosurgery and Psychiatry*.

[22] Ferreri F, Pauri F, Pasqualetti P, Fini R, Dal Forno G, Rossini PM (2003). Motor cortex excitability in Alzheimer’s disease: a transcranial magnetic stimulation study. *Annals of Neurology*.

[23] Pijnenburg YAL, Vd Made Y, Van Cappellen Van Walsum AM, Knol DL, Scheltens P, Stam CJ (2004). EEG synchronization likelihood in mild cognitive impairment and Alzheimer’s disease during a working memory task. *Clinical Neurophysiology*.

[24] Jiang ZY (2005). Study on EEG power and coherence in patients with mild cognitive impairment during working memory task. *Journal of Zhejiang University. Science. B*.

[25] Jiang ZY, Zheng LL (2006). Inter- and intra-hemispheric EEG coherence in patients with mild cognitive impairment at rest and during working memory task. *Journal of Zhejiang University. Science. B*.

[26] Zheng LL, Jiang ZY, Yu EY (2007). Alpha spectral power and coherence in the patients with mild cognitive impairment during a three-level working memory task. *Journal of Zhejiang University. Science B*.

[27] Zappoli R, Versari A, Paganini M (1995). Brain electrical activity (quantitative EEG and bit-mapping neurocognitive CNV components), psychometrics and clinical findings in presenile subjects with initial mild cognitive decline or probable Alzheimer-type dementia. *Italian Journal of Neurological Sciences*.

[28] Huang C, Wahlund LO, Dierks T, Julin P, Winblad B, Jelic V (2000). Discrimination of Alzheimer’s disease and mild cognitive impairment by equivalent EEG sources: a cross-sectional and longitudinal study. *Clinical Neurophysiology*.

[29] Koenig T, Prichep L, Dierks T (2005). Decreased EEG synchronization in Alzheimer’s disease and mild cognitive impairment. *Neurobiology of Aging*.

[30] Babiloni C, Binetti G, Cassetta E (2006). Sources of cortical rhythms change as a function of cognitive impairment in pathological aging: a multicenter study. *Clinical Neurophysiology*.

[31] Golob EJ, Irimajiri R, Starr A (2007). Auditory cortical activity in amnestic mild cognitive impairment: relationship to subtype and conversion to dementia. *Brain*.

[32] Moretti DV, Miniussi C, Frisoni GB (2007). Hippocampal atrophy and EEG markers in subjects with mild cognitive impairment. *Clinical Neurophysiology*.

[33] Moretti DV, Pievani M, Fracassi C (2009). Increase of theta/Gamma and Alpha3/Alpha2 ratio is associated with amygdalo-hippocampal complex atrophy. *Journal of Alzheimer’s Disease*.

[34] Moretti DV, Fracassi C, Pievani M (2009). Increase of theta/gamma ratio is associated with memory impairment. *Clinical Neurophysiology*.

[35] Moretti DV, Miniussi C, Frisoni G (2007). Vascular damage and EEG markers in subjects with mild cognitive impairment. *Clinical Neurophysiology*.

[36] Kramer JH, Reed BR, Mungas D, Weiner MW, Chui HC (2002). Executive dysfunction in subcortical ischaemic vascular disease. *Journal of Neurology Neurosurgery and Psychiatry*.

[37] Moretti DV, Babiloni C, Binetti G (2004). Individual analysis of EEG frequency and band power in mild Alzheimer’s disease. *Clinical Neurophysiology*.

[38] Klimesch W (1999). EEG alpha and theta oscillations reflect cognitive and memory performance: a review and analysis. *Brain Research Reviews*.

[39] Folstein MF, Folstein SE, McHugh PR (1975). “Mini mental state”. A practical method for grading the cognitive state of patients for the clinician. *Journal of Psychiatric Research*.

[40] Hughes CP, Berg L, Danziger WL (1982). A new clinical scale for the staging of dementia. *British Journal of Psychiatry*.

[41] Rosen WG, Terry RD, Fuld PA (1980). Pathological verification of ischemic score in differentiation of dementias. *Annals of Neurology*.

[42] Lawton MP, Brody EM (1969). Assessment of older people: self-maintaining and instrumental activities of daily living. *Gerontologist*.

[43] Albert M, Smith LA, Scherr PA, Taylor JO, Evans DA, Funkenstein HH (1991). Use of brief cognitive tests to identify individuals in the community with clinically diagnosed Alzheimer’s disease. *International Journal of Neuroscience*.

[44] Petersen RC, Smith GE, Waring SC, Ivnik RJ, Kokmen E, Tangelos EG (1997). Aging, memory, and mild cognitive impairment. *International Psychogeriatrics*.

[45] Portet F, Ousset PJ, Visser PJ (2006). Mild cognitive impairment (MCI) in medical practice: a critical review of the concept and new diagnostic procedure. Report of the MCI Working Group of the European Consortium on Alzheimer’s Disease. *Journal of Neurology, Neurosurgery and Psychiatry*.

[46] Geroldi C, Rossi R, Calvagna C (2006). Medial temporal atrophy but not memory deficit predicts progression to dementia in patients with mild cognitive impairment. *Journal of Neurology, Neurosurgery and Psychiatry*.

[47] Shulman KI (2000). Clock-drawing: is it the ideal cognitive screening test?. *International Journal of Geriatric Psychiatry*.

[48] Amodio P, Wenin H, Del Piccolo F (2002). Variability of trail making test, symbol digit test and line trait test in normal people. A normative study taking into account age-dependent decline and sociobiological variables. *Aging*.

[49] Basso A, Capitani E, Laiacona M (1987). Raven’s coloured progressive matrices: normative values on 305 adult normal controls. *Functional Neurology*.

[50] Spinnler H, Tognoni G (1987). Italian standardization and classification of neuropsychological tests. *Italian Journal of Neurological Sciences*.

[51] Carlesimo GA, Caltagirone C, Gainotti G (1996). The mental deterioration battery: normative data, diagnostic reliability and qualitative analyses of cognitive impairment. *European Neurology*.

[52] Caffarra P, Vezzadini G, Dieci F, Zonato F, Venneri A (2002). Rey-Osterrieth complex figure: normative values in an Italian population sample. *Neurological Sciences*.

[53] Radloff LS (1977). The CES-D scale: a self-report depression scale for research in the general population. *Applied Psychological Measurement*.

[54] Wahlund LO, Barkhof F, Fazekas F (2001). A new rating scale for age-related white matter changes applicable to MRI and CT. *Stroke*.

[55] Stam CJ, Van Der Made Y, Pijnenburg YAL, Scheltens P (2003). EEG synchronization in mild cognitive impairment and Alzheimer’s disease. *Acta Neurologica Scandinavica*.

[56] Seidenbecher T, Laxmi TR, Stork O, Pape HC (2003). Amygdalar and hippocampal theta rhythm synchronization during fear memory retrieval. *Science*.

[57] Narayanan RT, Seidenbecher T, Sangha S, Stork O, Pape HC (2007). Theta resynchronization during reconsolidation of remote contextual fear memory. *NeuroReport*.

[58] Montgomery SM, Buzsáki G (2007). Gamma oscillations dynamically couple hippocampal CA3 and CA1 regions during memory task performance. *Proceedings of the National Academy of Sciences of the United States of America*.

[59] Sauseng P, Klimesch W, Doppelmayr M, Hanslmayr S, Schabus M, Gruber WR (2004). Theta coupling in the human electroencephalogram during a working memory task. *Neuroscience Letters*.

[60] Bragin A, Jando G, Nadasdy Z, Hetke J, Wise K, Buzsaki G (1995). Gamma (40–100 Hz) oscillation in the hippocampus of the behaving rat. *Journal of Neuroscience*.

[61] Llinás RR, Ribary U, Jeanmonod D, Kronberg E, Mitra PP (1999). Thalamocortical dysrhythmia: a neurological and neuropsychiatric syndrome characterized by magnetoencephalography. *Proceedings of the National Academy of Sciences of the United States of America*.

[62] Klimesch W, Sauseng P, Hanslmayr S (2007). EEG alpha oscillations: the inhibition-timing hypothesis. *Brain Research Reviews*.

[63] Paré D, Collins DR, Pelletier JG (2002). Amygdala oscillations and the consolidation of emotional memories. *Trends in Cognitive Sciences*.

[64] Garolera M, Coppola R, Muñoz KE (2007). Amygdala activation in affective priming: a magnetoencephalogram study. *NeuroReport*.

[65] Young KA, Holcomb LA, Bonkale WL, Hicks PB, Yazdani U, German DC (2007). 5HTTLPR polymorphism and enlargement of the pulvinar: unlocking the backdoor to the limbic system. *Biological Psychiatry*.

[66] Ellison JM, Harper DG, Berlow Y, Zeranski L (2008). Beyond the “C” in MCI: noncognitive symptoms in amnestic and non-amnestic mild cognitive impairment. *CNS Spectrums*.

[67] Rozzini L, Vicini Chilovi B, Conti M (2007). Neuropsychiatric symptoms in amnestic and nonamnestic mild cognitive impairment. *Dementia and Geriatric Cognitive Disorders*.

[68] Johannsen P, Jakobsen J, Bruhn P, Gjedde A (1999). Cortical responses to sustained and divided attention in Alzheimer’s disease. *NeuroImage*.

[69] Berardi AM, Parasuraman R, Haxby JV (2005). Sustained attention in mild Alzheimer’s disease. *Developmental Neuropsychology*.

[70] Levinoff EJ, Saumier D, Chertkow H (2005). Focused attention deficits in patients with Alzheimer’s disease and mild cognitive impairment. *Brain and Cognition*.

[71] Tales A, Haworth J, Nelson S, Snowden RJ, Wilcock G (2005). Abnormal visual search in mild cognitive impairment and Alzheimer’s disease. *Neurocase*.

[72] Tales A, Snowden RJ, Haworth J, Wilcock G (2005). Abnormal spatial and non-spatial cueing effects in mild cognitive impairment and Alzheimer’s disease. *Neurocase*.

[73] Zalay OC, Bardakjian BL Simulated mossy fiber associated feedforward circuit functioning as a highpass filter.

[74] Frisoni GB, Galluzzi S, Bresciani L, Zanetti O, Geroldi C (2002). Mild cognitive impairment with subcortical vascular features: clinical characteristics and outcome. *Journal of Neurology*.

[75] Galluzzi S, Sheu CF, Zanetti O, Frisoni GB (2005). Distinctive clinical features of mild cognitive impairment with subcortical cerebrovascular disease. *Dementia and Geriatric Cognitive Disorders*.

[76] Steriade M, Llinas RR (1988). The functional states of the thalamus and the associated neuronal interplay. *Physiological Reviews*.

[77] Lopes da Silva FH, van Rotterdam A, Barts P, van Heusden E, Burr W (1976). Models of neuronal populations: the basic mechanisms of rhythmicity. *Progress in Brain Research*.

[78] Nunez PL, Wingeier BM, Silberstein RB (2001). Spatial-temporal structures of human alpha rhythms: theory, microcurrent sources, multiscale measurements, and global binding of local networks. *Human Brain Mapping*.

[79] Doiron B, Chacron MJ, Maler L, Longtin A, Bastian J (2003). Inhibitory feedback required for network oscillatory responses to communication but not prey stimuli. *Nature*.

[80] Nunez PL, Srinivasan R (2006). A theoretical basis for standing and traveling brain waves measured with human EEG with implications for an integrated consciousness. *Clinical Neurophysiology*.

[81] Szelies B, Mielke R, Kessler J, Heiss WD (1999). EEG power changes are related to regional cerebral glucose metabolism in vascular dementia. *Clinical Neurophysiology*.

[82] Leocani L, Locatelli T, Martinelli V (2000). Electroencephalographic coherence analysis in multiple sclerosis: correlation with clinical, neuropsychological, and MRI findings. *Journal of Neurology Neurosurgery and Psychiatry*.

[83] Goldensohn ES, Klass DW, Daly DD (1979). Use of EEG for evaluation of focal intracranial lesions. *Current Practice of Clinical Electroencephalography*.

